# Preparation and Characterization of Chitosan/Feldspar Biohybrid as an Adsorbent: Optimization of Adsorption Process via Response Surface Modeling

**DOI:** 10.1155/2014/370260

**Published:** 2014-01-23

**Authors:** Maryam Yazdani, Hajir Bahrami, Mokhtar Arami

**Affiliations:** ^1^Textile Engineering Department, Amirkabir University of Technology, Tehran, Iran; ^2^Department of Civil and Environmental Engineering, School of Science and Technology, Aalto University, P.O. Box 15200, 00076 Aalto, Finland

## Abstract

Chitosan/feldspar biobased beads were synthesized, characterized, and tested for the removal of Acid Black 1 dye from aquatic phases. A four-factor central composite design (CCD) accompanied by response surface modeling (RSM) and optimization was used to optimize the dye adsorption by the adsorbent (chitosan/feldspar composite) in 31 different batch experiments. Independent variables of temperature, pH, initial dye concentration, and adsorbent dose were used to change to coded values. To anticipate the responses, a quadratic model was applied. Analysis of variance (ANOVA) tested the significance of the process factors and their interactions. The adequacy of the model was investigated by the correlation between experimental and predicted data of the adsorption and the calculation of prediction errors. The results showed that the predicted maximum adsorption amount of 21.63 mg/g under the optimum conditions (pH 3, temperature 15°C, initial dye concentration 125 mg/L, and dose 0.2 g/50 mL) was close to the experimental value of 19.85 mg/g. In addition, the results of adsorption behaviors of the dye illustrated that the adsorption process followed the Langmuir isotherm model and the pseudo-second-order kinetic model. Langmuir sorption capacity was found to be 17.86 mg/g. Besides, thermodynamic parameters were evaluated and revealed that the adsorption process was exothermic and favourable.

## 1. Introduction

In today's industrial world, dyes are widely used in different areas, such as textiles, dye synthesis, and plastics. Due to huge consumption of water in these industries, water contamination by dyestuffs has been of major concern. Thus, the discharge of this dyeing effluent into our surrounding environment is a serious problem. In recent decades, dye removal from wastewaters has gained a great deal of attention because of their harmful impacts on aquatic organisms and human beings [1–3]. Among various techniques of removing dyes from water phases, adsorption is the preferred one which provides impressive results thanks to its capability of being used to eliminate different types of pollutants. Besides, to reduce the cost of wastewater treatment, many researchers have tried to find nonconventional alternative, so-called low-cost, adsorbents [1–4].

Thanks to its great number of amino (–NH_2_) and hydroxyl (–OH) sites, chitosan (CTS) which is the second most plentiful natural biopolymer provides a higher adsorption capacity and rapider adsorption rate for anionic species than many conventional adsorbents [5–8]. However, the market price of CTS is relatively high, and also its specific gravity should be enhanced for practical operation. Hence, numerous trials have been done to develop cheaper adsorbents derived from CTS; recently, various CTS-based composites, especially CTS-geological materials composites, have been tested as adsorbents for dyes and metal ions removal [7–10].

Locally available and environmentally friendly geological materials such as minerals, clay, and sandstone are being used as low-cost adsorbents for water and wastewater treatment purposes [11–13]. Among these materials, feldspars are the most abundant and important group of rock-forming minerals, constituting approximately around 60% of the earth crust. Due to their characteristics of natural abundance and potential adsorptive ability, feldspars are involved in inexpensive and environmental processes and especially in removing ionic pollutants from water and wastewater [14–16]. However, to our knowledge, studies on anionic dyes removal using feldspars are limited in the literature, and since several reports have recently presented that the modified geological materials provide higher adsorption capacity towards anionic components than their original, in this study, biobased CTS/feldspar composite was prepared for removing Acid Black 1 as a model to study adsorptive potential of the composite towards acid dyes.

In addition, because adsorption process is largely altered by operating parameters, such as initial dye concentration, pH, and temperature, to maximize the adsorption capacity, the process condition is in need of optimization [17]. In order to optimize operating variables with the minimum number of experiments, the experimental design techniques can be beneficial. One of the mostly used experimental design methodologies is response surface methodology (RSM). This method helps to determine linear, interaction, and quadratic effects of the variables and a prediction model for the response. Using RSM is more economical and less time consuming than using classical methods because it requires less number of experiments to be done [18, 19]. To the best of our knowledge, the efficiency of treatment and the effect of interaction of various parameters using RSM during dye adsorption with CTS/feldspar composite have not been reported. Thus, the present work is aimed at studying the adsorption of Acid Black 1 onto composite derived from feldspar and CTS through the experimental design and optimization of the process variables by applying RSM approach. The effects of initial dye concentration, adsorbent dose, solution pH, and temperature were optimized, and the isotherm, kinetic, and thermodynamic of the dye adsorption onto adsorbent were investigated.

## 2. Materials and Methods

### 2.1. Materials

Chitosan (degree of deacetylation: 85%; average molecular weight: 1000 kD) was supplied by Merck Enterprises Co. Acid Black 1 dye (AB1) was purchased from Ciba Ltd. and used without further purification. The chemical structure and characteristic of AB1 are shown in Table 1. All other chemicals were purchased from Merck (Germany). Local feldspar was provided from Hamedan, Iran.

### 2.2. Preparation of Chitosan/Feldspar Biobased Composite

Four grams of feldspar powder remained in 50 mL distilled water overnight. CTS solution was prepared by dissolving 1 g of CTS in 1% (v/v) aqueous acetic acid solution. The pH of the CTS solution was adjusted to 4 and followed by stirring at 60°C for 12 h. Subsequently, the pH of resulting solution was adjusted to 5 with 0.1 M NaOH solution and slowly added into feldspar suspension with vigorous stirring. The mixture was treated at 60°C for another 12 h to obtain CTS/feldspar composite. Next, the resulting solution was neutralized with 0.1 M NaOH which was added drop by drop, until precipitation occurred in the solution. The formed composite was filtered, washed with distilled water, and dried at 50°C for 24 h in vacuum oven. Finally, the sample was ground and sieved.

### 2.3. Characterization of Chitosan/Feldspar Biobased Composite

Local feldspar and CST/feldspar powder samples were analyzed by X-ray powder diffraction using a Philips Powder Diffractometer PW 1800. The IR spectra of the samples were recorded using a FT-IR spectrophotometer in the range 450–4000 cm^−1^ (FT-IR, Perkin-Elmer Spectrophotometer Spectrum One). Scanning electron microscopy technique was employed to study the morphology of the samples (SEM, Philips XL30 scanning electron microscope).

### 2.4. Response Surface Modeling and Experimental Design

Recently, RSM has been applied to optimize different process variables for dye removal purposes [17–19]. This technique aims to determine the optimum set of process variables and includes three steps of experimental design, response surface modeling, and process optimization. A standard RSM design, central composite design (CCD), has effectively been utilized to fit a second-order model. Generally, in a 2 level study, the CCD consists of 2^*n*^ factorial runs, 2*n* axial runs (indeed these 2*n* points fixed axially at a distance, *α*, from the center to produce the quadratic terms), and *n*_*c*_ center runs (2^*n*^ + 2*n* + *n*_*c*_), where *n* is the number of operating variables. The amount of *α* is calculated as 1/4th power to the number of factorial runs [20]. Thus, for the four parameters investigated at two levels, *α* = (2^4^)^1/4^.

Optimization process includes three significant steps: performing the statistically designed experiments, determining the coefficients in a mathematical model, and anticipating the response as well as checking the model adequacy [21]. The response model may be represented as

(1) $$\begin{array}{c} Y=\beta_{0}+\overset{n}{\underset{i=1}{\sum }}\beta_{i}X_{i}+\overset{n}{\underset{i=1}{\sum }}\beta_{ii}X_{i}^{2}+\overset{n-1}{\underset{i=1}{\sum }}\overset{n}{\underset{j=i+1}{\sum }}\beta_{ij}X_{i}X_{j}, \end{array}$$

where *Y* is the response, and the objective is to optimize the response (*Y*). *β*_0_ is the constant coefficient, is *β*_*i*_ the linear coefficients, *β*_*ii*_ is the quadratic coefficients, *β*_*ij*_ is the interaction coefficients, and *X*_*i*_ and *X*_*j*_ are the coded values of the independent process variables.

In our study, initial dye concentration (*C*_0_), adsorbent dose (*D*), initial pH (pH), and temperature (*T*) were four operating factors to investigate their effect on dye adsorption by the CTS/feldspar particles. The CCD technique was applied to determine the number of dye removal experiments to be done for the optimization of the variables. The statistical software “MiniTab” version 15 and “Design Expert 7.0.0” were used for CCD and the obtained data analysis. The experimental sequence was randomized to minimize the effects of the uncontrolled factors. For a design including four process variables each of them with two different levels, the number (*N*) of experiments should be calculated as *N* = (2^*n*^ + 2*n* + *n*_*c*_) = 2^4^ + (2 × 4) + 7 = 31. For statistical calculations, the variables *X* were coded as *X*_*i*_ according to the following equation:

(2) $$\begin{array}{c} X_{i}=\frac{X-X_{0}}{\delta X}, \end{array}$$

where *X*_0_ is the value of *X* at the centre point, and *δX* shows the step change. The selected independent variables with their limits, units, and notations are displayed in Table 2.

The response variable of the dye removal (*Y*) from the aqueous solutions using the CTS/feldspar can be written as

(3) Y=β0+β1XC0+β2XC02+β3XD+β4XD2+β5XT +β6XT2+β7XpH+β8XpH2+β9XC0XD +β10XC0XT+β11XC0XpH+β12XDXT +β13XDXpH+β14XTXpH,

where *X*_*C*_0__, *X*_*D*_, *X*_*T*_, and *X*_pH_ are the coded values of the variables. The coefficients of the model are estimated and applied to predict the response variable for different combinations of the coded values of process variables.

The statistical significance of the model was justified using analysis of variance (ANOVA) for polynomial model with 95% confidence level. Moreover, the correlation coefficient (*R* ^2^), the relative standard error of prediction (RSEP), and the root mean square error of prediction (RMSEP) were calculated using the experimental and anticipated values. The correlation coefficient represents the goodness of the model fit, and the RSEP and RMSEP values are used to assess the predictive ability of the model. The RSEP and RMSEP were calculated as follows:

(4) $$\begin{array}{c} \text{RSEP}=\sqrt{\frac{\sum_{i=1}^{N}{\left(y_{\text{pred},i}-y_{\mathrm{meas},i}\right)}^{2}}{\sum_{i=1}^{N}{\left(y_{\mathrm{meas},i}\right)}^{2}}}\times 100,\\ \text{RMSEP}=\sqrt{\frac{\sum_{i=1}^{N}{\left(y_{\text{pred},i}-y_{\mathrm{meas},i}\right)}^{2}}{N}}, \end{array}$$

where *y*_pred,*i*_ and *y*_meas⁡,*i*_ show the model predicted values and experimental data, and *N* indicates the number of experiments.

### 2.5. Adsorption Procedure

Adsorption experiments were carried out to investigate the effect of mentioned process variables on the dye removal process. To avoid systematic errors, the experiments were performed according to CCD at random. For fixing the minimum and maximum levels of each variable, pretrial experiments were also conducted. In the experiments, different weighted amounts of CTS/feldspar (4–12 g/L) were contacted with 50 mL of solutions with initial dye concentration of 25–125 mg/L. The solutions were made ready by dissolving known amount of AB1 in 1 L of distilled water. The stock solution was diluted as required to gain solutions of needed concentrations ranging between 25 and 125 mg/L, and their pH values were adjusted (3–11) using sulfuric acid and sodium hydroxide. Then, the prepared solutions were shaken in a thermocontrolled condition for 90 min at 200 rpm and at different temperatures (15–55°C). Other conditions such as the contact time were chosen based on the data that resulted from preliminary experiments. Finally, the equilibrated solutions were taken out, and the adsorbent was separated from them using a centrifuge. The concentration of the dye in the residual solution was determined using a UV-vis spectrophotometer at 618 nm. The amount of the dye (mg/g) adsorbed on the biosorbent was calculated as

(5) $$\begin{array}{c} q_{e}=\frac{\left(C_{0}-C_{e}\right)V}{M}, \end{array}$$

where *C*_0_ and *C*_*e*_ represent the initial and final concentrations (mg/L) of AB1 in solution, respectively. The *M* and *V* are the adsorbent weight (g) and the solution volume (L), respectively. The experimental conditions and the dye adsorption measured through the experiments are given in Table 3.

### 2.6. Validation of the Model

The validation of the model was verified through performing some sets of different mixtures of the operating parameters each of them in their experimental ranges. After having turned the operating variables into their related coded values by (2), corresponding response variable (*Y*) was computed using the response surface model. Batch experiments were carried out to find out the amount of adsorption under fixed conditions of all the operating variables for defined validation sets. Also, the experimental results and the model predicted data of the adsorption for validation sets were applied to calculate the correlation coefficient (*R* ^2^), the relative error of prediction (RSEP), and the root mean square error of prediction (RMSEP).

### 2.7. Process Optimization

The optimal values of the variables were obtained by analyzing response surface plots and also by using the developed quadratic model. Lastly, the optimal values were more proved by actually performing trails at the optimum conditions.

## 3. Results and Discussion

### 3.1. Characterization of the Biobased Sorbent

#### 3.1.1. IR Analysis of CTS/Feldspar Biobased Composite

**Figure 1:**
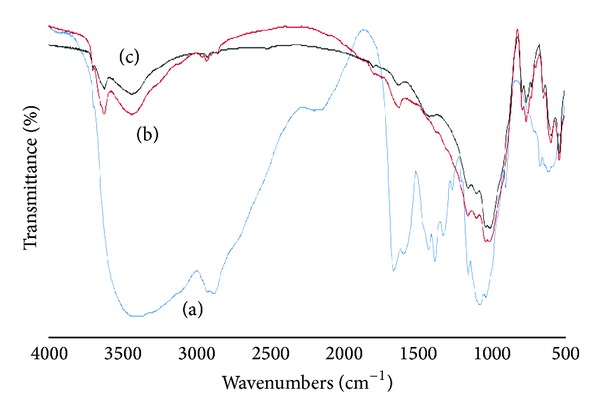
IR spectra of the chitosan (a), the CTS/feldspar (b), the feldspar (c).

Figure 1 indicates the IR spectra obtained from feldspar, chitosan, and the CTS/feldspar composite, in the 4000–450 cm^−1^ wavenumber range. As it can be seen, the absorption band of the CTS/feldspar at 3435 and 3624 cm^−1^ has been enhanced, which demonstrates the vibration bands in CTS (O–H and N–H stretching) overlap with the bands of feldspar (–OH stretching of H_2_O). The bands at 2923 and 2849 cm^−1^ related to the intercalated CTS (C–H stretching on methyl and methylene groups, resp.) are seen in the spectra of the CTS/feldspar composite. Also, the bands at 1375 cm^−1^ related to the intercalated CTS (C–H bending on methylene groups) can be seen in the spectra of the biobased composite. The band at 1530 cm^−1^ attributed to the deformation vibration of the protonated amine group of CTS has become a little stronger in the spectra of CTS/feldspar. The intensity of the band at 1635 cm^−1^ of the CTS/feldspar spectra has increased, indicating the first NH–CO group stretching vibration of CTS overlap with –OH bending vibration of H_2_O of the feldspar. In addition, corresponding to the vibration bands of the silicate, the bands at 1094, 1035, and 760 cm^−1^ remain unaffected in the biobased composite [7, 22].

#### 3.1.2. X-Ray Diffraction Analysis of Biobased Composite

**Figure 2:**
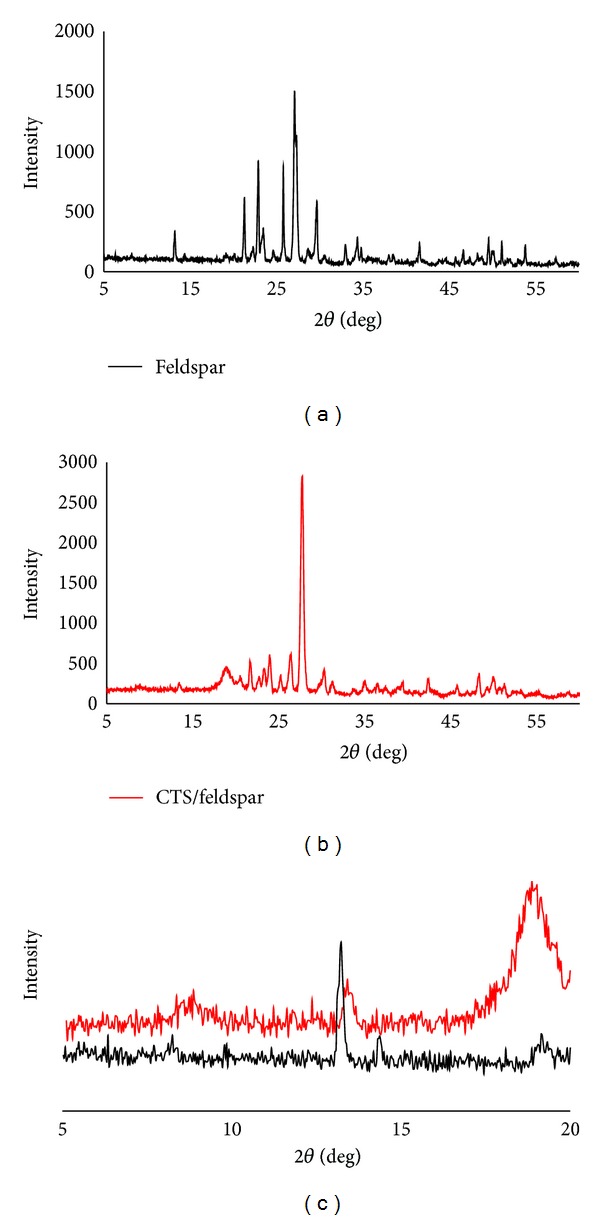
XRD patterns of (a) feldspar and (b) CTS/feldspar biobased composite.

The chemical composition of feldspar is given in Table 4. The major constituents of feldspar are silica and alumina. In addition, the mineralogical composition of the feldspar was determined from X-ray diffractogram (Figure 2(a)), and the following mineral phases were identified: oligoclase, quartz, illite, and calcite.

Figures 2(a) and 2(b) illustrate the XRD patterns of feldspar and CTS/feldspar powders, respectively. The XRD pattern of feldspar shows typical diffraction peaks at 2*θ* = 13.25 and 14.40°, whereas, after incorporating with Chitosan, these peaks shift to lower angles and even disappear. Also, after incorporating with CTS, the peak at 2*θ* = 14.40°, which is corresponding to a basal spacing of 6.14 Å, shifts to a lower angle. Besides, it can be seen in the XRD pattern of CTS/feldspar that there is a diffraction peak at 13.45 attributing to a basal spacing of 6.6 Å, which means the intercalation has occurred. The XRD pattern of the feldspar also shows a reflection peak at about 2*θ* = 13.25°. After incorporating feldspar within CTS biopolymer, this peak is substituted by a new weakened broad peak at around 2*θ* = 8–10 Å. The movement of the basal reflection of feldspar to a lower angle shows the formation of an intercalated structure, while the peak broadening and intensity decrease likely suggest the disordered intercalated in CTS/feldspar composite [23, 24].

#### 3.1.3. SEM Image Analysis of CTS/Feldspar Biobased Composite

**Figure 3:**
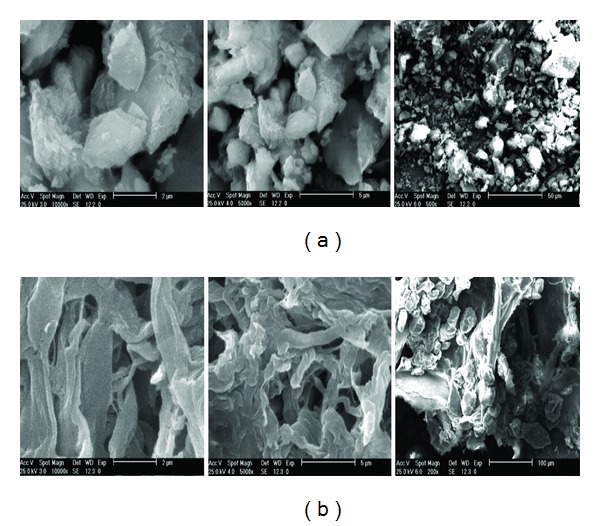
SEM images of raw feldspar (a) and CTS/feldspar biobased composite (b).

The morphologies of feldspar and CTS/feldspar samples were studied by SEM.  Figure 3 displays different views of these materials. As can be observed, the morphologies of feldspar (Figure 3(a)) and CTS/Feldspar (Figure 3(b)) are so different. In feldspar microphotographs, the small particles with a nonporous surface can be observed, while in CTS/feldspar it is seen that the feldspar particles are surrounded by an irregularly shaped CTS net. Also, the introduction of CTS has led to a larger particle size and a coarse porous surface. These large particles and numerous cavities are more convenient for the penetration of the dye molecules into CTS/feldspar and would result in an enhancement in the adsorption capacity of AB1 on CTS/feldspar composite.

### 3.2. Development of Quadratic Model Equation

In this work, CCD method was used to study the adsorption of AB1 onto the CTS/feldspar. A quadratic model was chosen to explore the mathematical relation between the response variable (adsorption amount) and aforementioned independent variables. Based on the RSM results (Table 5), a regression model equation was developed between the response variable (*Y*) and the corresponding coded values (*X*_*C*_0__, *X*_*D*_, *X*_pH_, and *X*_*T*_) of the independent variables, and the most fitted relationship resulted as

(6) Y (mg/g)=3.11+0.61XC0+0.04XC02+0.15XD −0.06XD2−2.25XpH+1.35XpH2−1.09XT +0.12XT2+0.19XC0XD−0.58XC0XpH −0.28XC0XT+0.16XDXpH +0.35XDXT+0.10XpHXT.

Equation (7) was employed to analyze the effect of the independent variables on adsorption amount (*Y*). Table 5 indicates the results of statistical analysis. A higher *F* value results in a greater significance of the related variable. Also, *P* values less than 0.05 for model variables indicate that model variables are significant [17, 21]. As can be seen in Table 5, the linear terms of three variables (pH, temperature, and dye concentration) and only the quadratic term of pH variable are statistically significant (*P* < 0.05); also among the interactive terms, the interactions of pH and dye concentration variables are significant. Thus, these results revealed that pH, temperature, and dye concentration present the most significant effects on the adsorption process. The sign of regression coefficients is also important, which gives the direction of variable effect on the response. A positive coefficient sign indicates a synergistic effect, whereas a negative sign displays an antagonistic effect. On the one hand, two independent variables (pH and temperature) as well as the interactive term of pH and dye concentration (pH × *C*) showed negative significant relationship with the dye removal. On the other hand, dye concentration and the quadratic term of pH exhibited positive significant effect on dye adsorption.

**Figure 4:**
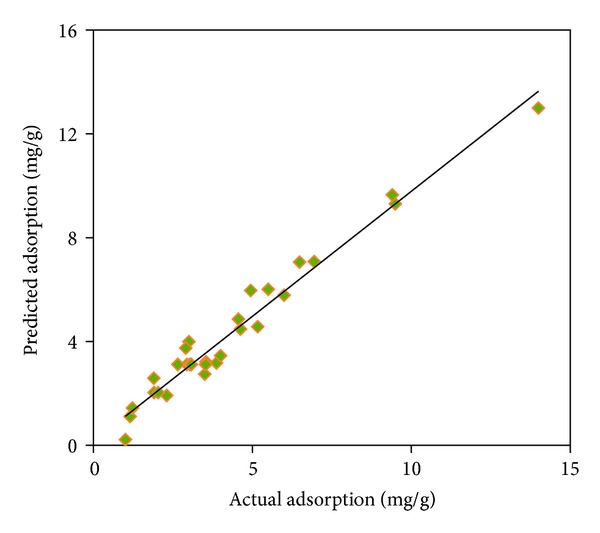
Actual (mg/g) and predicted plot of the adsorption of the AB1 for the model development sets.

The experimental data obtained from the adsorption trails and the values predicted by the response surface model are presented in Figure 4. The high correlation (*R* ^2^ = 0.96) between the experimental data and the predicted amounts of the response and also the low RMSEP (0.52) and RSEP (1.02) support the adequacy of the developed quadratic model in predictive uses.

### 3.3. Validation of the Model

**Figure 5:**
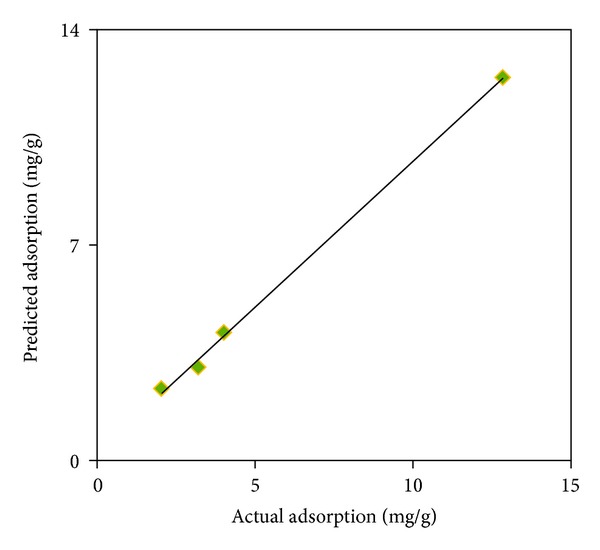
Actual and predicted plot of the adsorption (mg/g) of the AB1 for the model validation sets.

The model validation was carried out through performing four experiment sets with different combinations of the process variables all of which within their respective experimental range. Then, related response values (*Y*, mg/g) were determined using the coded values of the process variables and (7). Moreover, the experimental data resulted from these four sets combined with their predicted values of the model were used to calculate the correlation coefficient, RMSEP, and RSEP values. The experimental and model predicted results are indicated in Table 6. A high correlation (*R* ^2^ = 0.99) between the experiments and predicted results of the response factor (Figure 5) and also fairly low RMSEP (0.49) and RSEP (0.69) values demonstrate the adequacy of the model to predict the response variable for the validation data sets.

### 3.4. Response Surface Plotting

#### 3.4.1. Effect of Temperature and pH on Adsorption Process

**Figure 6:**
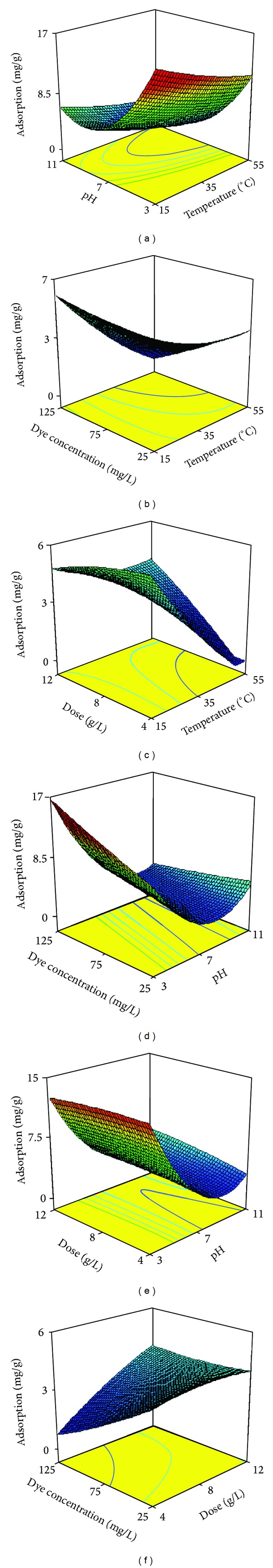
The 3D plots showing effect of (a) pH and temperature, (b) temperature and initial dye concentration, (c) temperature and adsorbent dose, (d) pH and initial dye concentration, (e) pH and adsorbent dose, and (f) initial dye concentration and adsorbent dose on the adsorption of AB1.

According to the literature, the role of pH and temperature variables is very significant in adsorption process; indeed, they are considered to be the most influencing variables in adsorption process.  Figure 6(a) is 3D response surface plot displaying the combined effect of temperature and pH on AB1 removal. As can be seen, an increase in the temperature leads to a decrease in the adsorption rate of the dye; this effect demonstrates that the adsorption of AB1 onto the surface of sorbent particles is favored at lower temperatures and is controlled by an exothermic process. One reason for this trend may be partly due to weakening of attractive forces between the dye molecules and the surface of particles [25]. This diagram also reveals that the adsorption extremely depends on the solution pH. The rate of adsorption in the experimental pH range 3–11 decreases with an increase in the pH of the solution. Similar results have also been observed earlier by other researchers [17, 21, 25, 26]. This adsorptive behavior of the anionic dye onto the prepared composite may be hidden in the nature of the sorbent surface. At lower pH, the more available protons on its surface increase electrostatic attractions between negatively charged dye anions and positively charged adsorption sites; consequently the amount of dye adsorption would increase. Thus, thanks to the electrostatic interaction among abundant number of –NH_3_^+^ sites of CTS/feldspar and AB1 anions in acidic medium, a higher adsorption capacity can be obtained. On the other hand, when pH of the solution is increased, the positive charges at the interface between the adsorbent and solution would decline; so, the adsorbent surface emerges negatively charged. Therefore, a lower adsorption at higher pH may result by the ionic repulsion between the negatively charged surface of adsorbent and dye anions [6, 25, 26]. A maximum adsorption amount of 15.59 mg/g was achieved at constant initial dye concentration (75 mg/L) and adsorbent dose (8 g/L).

#### 3.4.2. Effect of Temperature and Dye Concentration on Adsorption Process

The combined effect of initial dye concentration and temperature on the dye adsorption onto CTS/feldspar is exhibited in Figure 6(b). It is obvious that an increase in the process temperature would decline the adsorption rate. On the contrary, equilibrium uptake enhances with increasing of initial dye concentration. To explain this phenomenon, it can be said that in a constant condition, when the concentration of the adsorbate in the solution becomes bigger, the active sites of the adsorbent will be surrounded by much more molecules of it, and thereby the process of adsorption will perform more sufficiently. Thus in this case, the values of *q*_*e*_ increased with an enhancement in initial concentration of the dye. About the bigger adsorption capacity in the lower temperature range, it may be due to a tendency for the dye molecules to escape from the solid phase to the liquid phase with an increase in the solution temperature. This decrease of adsorption amount with increasing process temperature indicates that the adsorption of AB1 onto CTS/feldspar is exothermic in nature. Hence, the adsorption process may be physical. Previously, a similar trend has been observed [27, 28]. A maximum dye removal (8.27 mg/g) resulted at constant pH (7) and dose (8 g/L).

#### 3.4.3. Effect of Temperature and Adsorbent Dose on Adsorption Process

The plot for the interactive effect of temperature and adsorbent dose on the dye uptake by the biobased composite is represented in Figure 6(c). As can be seen, the adsorption amount decreases with an increase in solution temperature. In addition, at low temperatures, a declining trend is observed with increasing the CTS/feldspar dose. In a solution of a constant dye concentration, an increasing dose may lead to declining the number of adsorbate molecules in the solution, and as a result the flux from the aqueous medium to the surface of adsorbent would be reduced. Singh et al. [17, 29] also gave details of a decrease in dye uptake with increasing adsorbent dose. However, it should be mentioned that the trend is reversed at higher temperature, exhibiting an increasing uptake with increasing sorbent dose. A maximum dye removal (6.63 mg/g) was achieved at constant pH (7) and dye concentration (75 mg/L).

#### 3.4.4. Effect of pH and Initial Dye Concentration on Adsorption Process

Figure 6(d) represents the diagram for the combined effect of pH and initial dye concentration on the adsorptive behavior of AB1 onto the CTS/feldspar. While the dye removal increases with an increase in initial dye concentration, it decreases with an enhancement in the solution pH as in the cases discussed earlier. Decreasing rate in dye uptake with increasing pH may be related to the similar charges (negative) on the surface of the biosorbent and on the dye molecules in higher pH. A maximum dye uptake (16.71 mg/g) resulted at constant temperature (35°C) and adsorbent dose (8 g/L).

#### 3.4.5. Effect of pH and Adsorbent Dose on Adsorption Process

As seen in Figure 6(e), the response surface diagram for the interactive effect of the adsorbent dosage and the initial pH says that the dye adsorption by the CTS/feldspar extremely depends on the pH of the solution. AB1 removal at lower pH is higher than the dye removal at higher pH in the experimental pH range. As has already been mentioned, at the lower pH, there are a great number of protons in the experiment solution. Conversely, at the higher pH, there are a considerable number of OH^−^ in the aqueous medium. Therefore, the adsorbent can provide more positive charges in acidic medium rather than alkaline medium, and since the structure of the dye is anionic, removing dye from the acidic solution will be easier. In addition, it can be seen that the change of adsorbent dose does not have a special effect on dye removal efficiency even in different pH. A maximum dye removal (13.11 mg/g) was obtained at constant temperature (35°C) and initial dye concentration (75 mg/L).

#### 3.4.6. Effect of Initial Dye Concentration and Adsorbent Dose on Adsorption Process

Figure 6(f) displays the combined effect of initial dye concentration and adsorbent dose. It is clear that at lower dye concentrations, there is no significant change in dye removal through increasing sorbent dose. On the other side, at higher dye concentrations, the dye uptake increases with increasing dose. This trend can be explained in this way that at lower dye concentrations there are not enough dye molecules in the solution to occupy all the available binding sites; thus an increase in the sorbent dose would not affect the amount of adsorption. On the contrary, at higher concentrations, the adsorption will be relatively higher due to presence of enough dye molecules in the solution to be adsorbed by relatively more active binding sites provided by increasing sorbent dose. At constant temperature (35°C) and solution pH (7), a maximum dye uptake of 5.15 mg/g was reached.

### 3.5. Process Optimization

**Figure 7:**
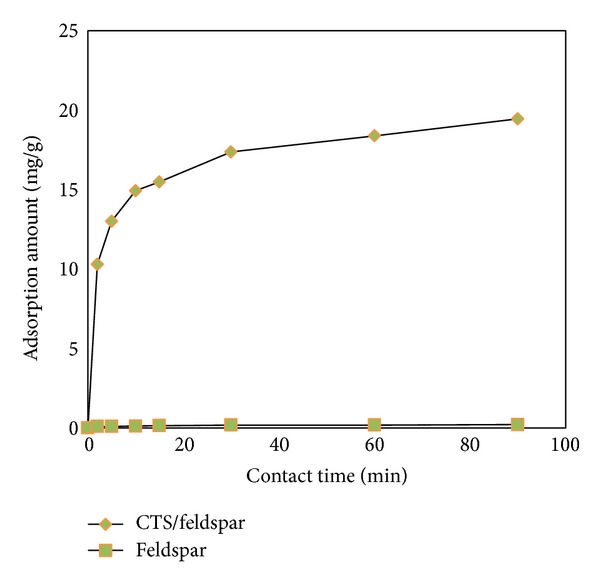
Effect of the adsorption time on adsorption capacity of feldspar and CTS/feldspar for AB1; experimental conditions: dye concentration, 125 mg/L; pH 3.0; temperature, 15°C.

The main objective of the present work was to reach the most effective values of process variables to optimize the AB1 adsorption by the CTS/feldspar composite from aqueous medium; this aim was fulfilled using the quadratic model within the studied experimental range of the considered parameters. The optimization determination suggested the optimum values of considered variables as pH 3, temperature 15°C, initial dye concentration 125 mg/L, and adsorbent dose 0.2 g/50 mL to gain the maximum dye uptake (21.63 mg/g) from the solution. Correspondingly, the experimental amount of the dye removal under the optimal condition of the process parameters was obtained as 19.85 mg/g. It should be mentioned that the experimental amount of AB1 adsorption onto feldspar in optimum values of process variables was determined as 0.2 mg/g. It is evident in Figure 7 that the adsorption amount of AB1 onto feldspar is less than the adsorption amount of AB1 onto CTS/feldspar. This suggests that this anionic dye has low tendency to feldspar particles, and its blending with chitosan results in an increase in the dye adsorption onto the hybrid.

### 3.6. Equilibrium Studies

**Figure 8:**
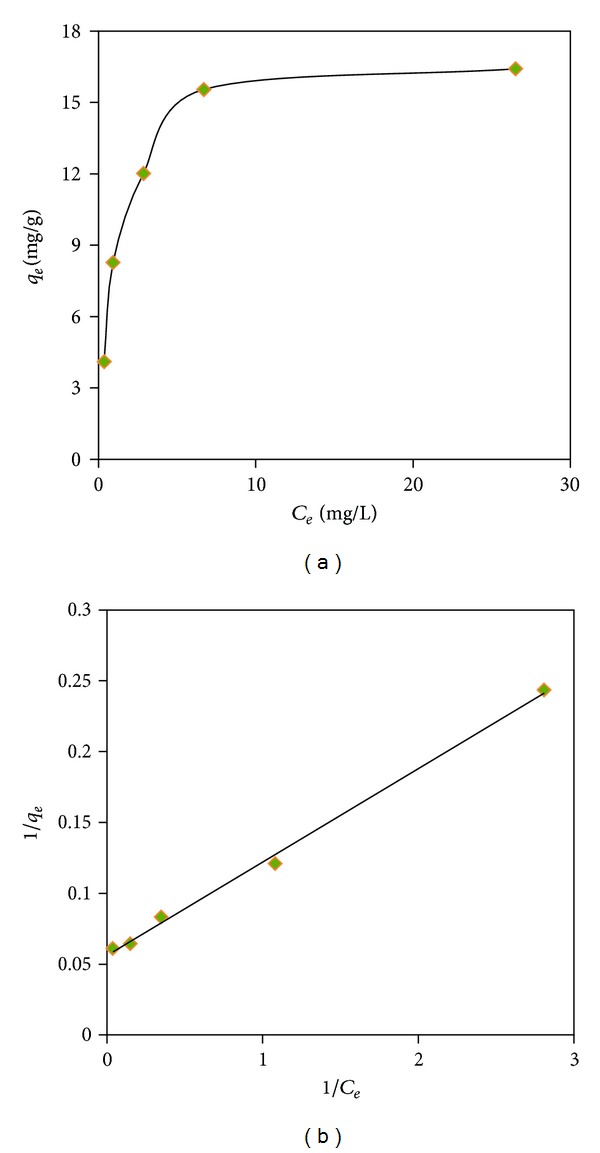
Plots of (a) *q*_*e*_ versus *C*_*e*_ and (b) the linearized form of the Langmuir model for the adsorption of AB1 onto the CTS/feldspar composite; pH 3 and 15°C.

The Langmuir isotherm model was used to evaluate the adsorption capacity of AB1 (mg/g) onto the prepared CTS/feldspar biosorbent. As known, this isotherm model is based on some basic assumptions including the following: first, adsorption happens on specific homogeneous sites; second, the dye molecule occupies the site; third, the adsorbent has a finite capacity for the adsorbate which means at equilibrium, a saturation level is attained and further adsorption could not occur; and finally, all sites are identical and energetically equivalent [30]. The linear equation of the model is written as

(7) $$\begin{array}{c} \frac{1}{q_{e}}=\frac{1}{q_{\max\;}K_{L}C_{e}}+\frac{1}{q_{\max}}, \end{array}$$

where *q*_max⁡_ (mg g^−1^) is the maximum capacity of the adsorbate to create a complete monolayer on the surface of the adsorbent; *K*_*L*_ (Lmg^−1^) is the Langmuir constant related to the adsorption heat. By plotting 1/*q*_*e*_ versus 1/*C*_*e*_, it is possible to obtain the *q*_max⁡_ and *K*_*L*_ values. The experimental adsorption isotherm graph is given in Figure 8(a). The linearized form of the isotherm model (Figure 8(b)) resulted in a high correlation coefficient (*R* ^2^ = 0.99). This high correlation coefficient states that the dye adsorption follows the Langmuir model of adsorption. Besides, the sorption capacity (*q*_max⁡_) of the CTS/feldspar and the second Langmuir constant *K*_*L*_ related to the free energy adsorption were found to be 17.857 mg/g and 0.848 L·mg^−1^ at 25°C and pH 3, respectively.

### 3.7. Kinetics of Adsorption

Pseudo-first-order and pseudo-second-order kinetic models were employed for kinetic studies. The pseudo-first-order equation is a simple kinetic model describing the adsorption process which has been suggested by Lagergren [31] for the adsorption of solid/liquid systems; its formula is given as

(8) $$\begin{array}{c} \log\left(q_{e}-q_{t}\right)=\log\left(q_{e}\right)-\frac{k_{1}}{2.303}t, \end{array}$$

where *k*_1_ is the pseudo-first-order rate constant (min^−1^) and *q*_1_ and *q*_*t*_ are the amounts of dye adsorbed (mg/g) at equilibrium and at time *t* (min).

Pseudo-second-order model is based on the adsorption equilibrium capacity and is expressed as [32]

(9) $$\begin{array}{c} \frac{t}{q_{t}}=\frac{1}{k_{2}q_{e}^{2}}+\frac{t}{q_{e}}, \end{array}$$

where *k*_2_ (g/mg min) is the rate constant of the pseudo-second-order adsorption. The *k*_1_, *k*_2_, *q*_1_, *q*_*e*_, and correlation coefficients, *r*_1_^2^ and *r*_2_^2^, of AB1 adsorption under different conditions were calculated from the plots of experimental data and are given in Table 7. The correlation coefficients (*r*_1_^2^) for the pseudo-first-order kinetic model are between 0.774 and 0.927, and the correlation coefficients (*r*_2_^2^) for the pseudo-second-order kinetic model are between 0.997 and 1. Thus, probably the adsorption process of AB1 onto CTS/feldspar is not a pseudo-first-order reaction, but it fits the pseudo-second-order kinetic model. Also, these results suggest that the adsorption rate of AB1 depends on the dye concentration at the surface of absorbent. Besides, the pseudo-second-order model anticipates the behavior over the whole experimental range, which demonstrates the validity of the model.

### 3.8. Thermodynamic Studies

Thermodynamic parameters including free energy change (Δ*G*°), enthalpy change (Δ*H*°), and entropy change (Δ*S*°) were estimated using the following equations [33, 34]:

(10) $$\begin{array}{c} K_{D}=\frac{C_{Z}}{C_{S}},\\ \Delta G=-RT\ln K_{D},\\ \ln K_{D}=\frac{\Delta S}{R}-\frac{\Delta H}{RT}, \end{array}$$

where *C*_*S*_ and *C*_*Z*_ (mg/L) are the equilibrium concentrations of the dye on the composite and in the solution, respectively; *K*_*D*_ is the equilibrium constant; *T* is the solution temperature (*K*_*D*_); and *R* is the gas constant (8.314, JK^−1 ^mol^−1^).

The Δ*G*° is used to explore the spontaneity of the adsorption process; the higher the negative value, the more energetically favorable process. The Δ*G*° values in Table 8 suggest that AB1 adsorption by CTS/feldspar is spontaneous and is favorable at lower temperatures [33]. Δ*H*° and Δ*S*° values for all cases are negative, indicating adsorption processes are exothermic and randomness decreases at the solid-solution interface during adsorption [34].

## 4. Conclusion

In this study, synthesis and characterization of a biobased chitosan/feldspar composite were intended. Besides, the adsorption capacity of the prepared composite and the optimization of the operating variables to maximize the AB1 adsorption onto it were studied. The RSM model based on full factorial CCD was applied to determine the effect of four selected independent variables on the dye uptake by the CTS/feldspar biosorbent from the aqueous medium. The quadratic model used for response surface analysis determined the pH, temperature, and dye concentration as the most significant parameters affecting the dye removal process. The optimum adsorption conditions ascertained for the new biosorbent were pH 3, temperature 15°C, adsorbent dose 0.2 g/50 mL, and dye concentration 125 mg/L. The experimental value of dye adsorption (19.85 mg/g) was observed to fit fairly with the model predicted value (21.63 mg/g). Furthermore, the adsorption kinetics obeys the pseudo-second-order model, and the adsorption isotherm follows the Langmuir model. Thermodynamic parameters, including Δ*G*°, Δ*H*°, and Δ*S*°, were determined and revealed that the adsorption process was exothermic and spontaneous. Therefore, as an environmentally friendly biobased composite material, CTS/feldspar can be a propitious sorbent for water and wastewater treatment purposes.

## Figures and Tables

**Table 1:** Characteristic and chemical structure of AB1.

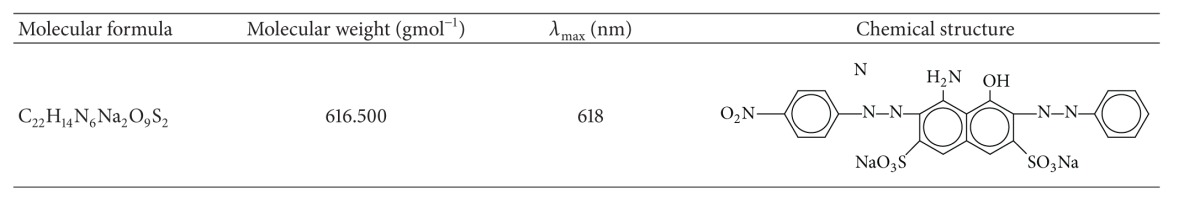

**Table 2:** Process control variables and their limits.

Variable	Unit	Notation	Limits				
			−2	−1	0	1	2
Initial dye concentration	MgL^−1^	*C*_0_	25	50	75	100	125
Dose	gL^−1^	*D*	4	6	8	10	12
pH		pH	3	5	7	9	11
Temperature	°C	*T*	15	25	35	45	55

**Table 3:** Central composite design (CCD) for the four independent variables and corresponding dye adsorption (mg/g).

CCD term	Number of experiments	Coded values of process variables				Adsorption (mg/g)
		*C*_0_	*D*	pH	*T*	
Factorial (2^*n*^)	16	−1, +1	−1, +1	−1, +1	−1, +1	1–9.5
Axial (2*n*)	8	−2, 0, +2	−2, 0, +2	−2, 0, +2	−2, 0, +2	1.23–14
Center (*n*_*c*_)	7	0	0	0	0	2.65–3.53

**Table 4:** Chemical analysis of feldspar.

Constituent	Value (Wt.%)
SiO_2_	64.6
Al_2_O_3_	21.2
Fe_2_O_3_	0.30
TiO_2_	0.86
Na_2_O	5.76
K_2_O	0.85
CaO	4.00
MgO	0.41

**Table 5:** Analysis of variance (ANOVA) for response surface quadratic model for adsorption of AB1.

Term	Coefficient	Sum of squares	DF	Mean squares	*F* value	*P* value	Remarks
Model	—	222.56	14	15.90	30.52	0.000	Significant
*X*_pH_	−2.25	121.82	1	121.82	233.91	0.000	Significant
*X*_*T*_	−1.09	28.31	1	28.31	54.37	0.000	Significant
*X*_*D*_	0.15	0.50	1	0.50	0.97	0.340	
*X*_*C*_0__	0.61	8.97	1	8.97	17.22	0.001	Significant
*X*_pH_^2^	1.35	51.75	1	51.75	99.36	0.000	Significant
*X*_*T*_^2^	0.12	0.44	1	0.44	0.84	0.372	
*X*_*D*_^2^	−0.06	0.10	1	0.10	0.19	0.667	
*X*_*C*_0__^2^	0.04	0.038	1	0.04	0.073	0.790	
*X*_pH_ *X*_*T*_	0.10	0.16	1	0.16	0.31	0.583	
*X*_pH_ *X*_*D*_	0.16	0.41	1	0.41	0.80	0.386	
*X*_pH_ *X*_*C*_0__	−0.58	5.41	1	5.41	10.38	0.005	Significant
*X*_*T*_ *X*_*D*_	0.35	1.96	1	1.96	3.77	0.070	
*X*_*T*_ *X*_*C*_0__	−0.28	1.27	1	1.27	2.43	0.139	
*X*_*D*_ *X*_*C*_0__	0.19	0.58	1	0.58	1.12	0.305	
Residual		8.33	16	0.52	—	—	
Lack of fit		7.73	10	0.77	7.66	0.011	Significant
Pure error		0.61	6	0.10			
Correl. total		230.90	30				
Intercept	3.11		1			0.000	Significant

**Table 6:** The experiment (*E*) and the coded (*C*) values of the independent process variables in the validation model and corresponding values of the response variable.

Exp. number	pH		Temperature (°C)		Dose (g/L)		Dye concentration (mg/L)		Adsorption (mg/g)	
	*E*	*C*	*E*	*C*	*E*	*C*	*E*	*C*	Exp.	Pred.
1	4	−1.5	20	−1.5	5	−1.5	75	0	12.65	12.44
2	10	1.5	25	−1	11	1.5	100	1	4.021	4.148
3	8	0.5	40	0.5	9	0.5	100	1	2.140	2.335
4	6	0.5	30	−0.5	5	−1.5	25	−2	3.205	3.028

**Table 7:** Kinetics parameters for the adsorption of AB1.

Dye concentration (mg L^−1^)	Pseudo-first-order *t* = 0–90 min			Pseudo-second-order *t* = 0–90 min		
	*K*_1_ (min^−1^)	*q*_1_ (mg g^−1^)	*r*_1_^2^	*K*_2_ (g/mg^−1^ min^−1^)	*q*_*e*_ (mg g^−1^)	*r*_1_^2^
25	0.071	1.154	0.777	0.240	4.167	1
50	0.088	2.051	0.774	0.140	8.333	0.999
75	0.046	6.383	0.892	0.022	12.500	0.997
100	0.046	9.506	0.927	0.014	16.129	0.997
125	0.042	9.12	0.861	0.013	17.241	0.997

**Table 8:** Thermodynamic parameters for the adsorption of AB1 on CTS/feldspar (pH, 3; Co, 125 mgL^−1^).

Temperature (°C)	Δ*G*° (kJ/mol)	Δ*H*° (kJ/mol)	Δ*S*° (J/K mol)
15	−3.60	−24.15	−71.50
25	−2.72		
35	−2.13		
45	−1.65		
55	−0.56		
